# Burnout in Gastroenterology Unit Nurses

**DOI:** 10.3390/ijerph17093115

**Published:** 2020-04-30

**Authors:** Andreea Iulia Socaciu, Razvan Ionut, Maria Barsan, Andreea Petra Ungur, Armand Gabriel Rajnoveanu

**Affiliations:** Department of Occupational Health, Iuliu Hatieganu University of Medicine and Pharmacy, 400012 Cluj-Napoca, Romania; ionut.razvan@umfcluj.ro (R.I.); maria.opritoiu@umfcluj.ro (M.B.); andreea.ladaru@umfcluj.ro (A.P.U.); armand.rajnoveanu@umfcluj.ro (A.G.R.)

**Keywords:** burnout, gastroenterology, registered nurses, work-related stress, preventative medicine

## Abstract

(1) Background: Burnout syndrome is a significant problem in nursing professionals but may be dependent on the type of care that they provide. The objectives of our study are to identify and explore risk factors associated with burnout among gastroenterology nurses. Identifying the risk factors involved is an essential element for prevention programs. (2) Methods: We performed an analytical descriptive cross-sectional study. Burnout was measured using an adapted version of the Maslach Burnout Inventory (MBI) questionnaire. Strength of association between burnout scores and risk factors was calculated using Fischer’s exact test; (3) Results: Our subjects were all female nurses. Work-related risk factors, such as an increased workload and a large number of night shifts have been associated with burnout in nurses, together with a lack of physical activity. We found no significant associations with sociodemographic factors; (4) Conclusions: Gastroenterology nurses are affected by high levels of emotional exhaustion. Work-related risk factors and a sedentary lifestyle result in a greater prevalence of burnout. In this category of healthcare workers, preventive actions are needed. The physical activity outside work could be a protective factor for burnout, and an exercise program could contribute to the effectiveness of well-established burnout intervention programs.

## 1. Introduction

Burnout syndrome is a significant problem in nursing professionals but may be dependent on the type of care that they provide. Each medical service has different characteristics that can influence burnout levels [1]. Chronic occupational burnout can be associated with reduced job performance, cognitive impairment such as memory and attention, reduced tolerance for stress associated with sick-leave and thoughts of quitting, and also coronary heart disease and mental health problems. Lapses in attention increase the risk of serious consequences such as medication errors and other essential patient safety issues [2].

The levels of burnout among medical personnel, especially nurses, have a certain variability. As shown in some studies, following the Maslach Burnout Inventory (MBI burnout assessment, that describes burnout as having three dimensions (emotional exhaustion (EE), depersonalization (DP) and low personal accomplishment (PA)) [3,4], the prevalence of emotional exhaustion is around 30 percent, in oncology and emergency nurses, while depersonalization occurs in 15 percent of the oncology nurses and 36 percent in emergency nurses [5,6]. According to our knowledge, there are no studies to date concerning burnout in gastroenterology (GE) nurses. This represents a research gap in this field. There are several studies that report moderate to high levels of emotional exhaustion in up to 32–63 percent of gastroenterologists, varying based on years of experience, with an average of 37 percent [7,8,9,10,11], in a context in which burnout occurs in approximately 40–45 percent of all physicians [12,13]. Gastroenterology is a unique medical specialty, where the basic activities are very similar to internal medicine or primary care, but most gastroenterology physicians, assisted by nurses, perform various procedures daily. These include diagnostic and therapeutic colonoscopy and upper endoscopy, endoscopic retrograde cholangiopancreatography, endoscopic ultrasonography, colon, liver, and stomach biopsies, treatment of variceal bleeds, and other upper digestive tract hemorrhages. These invasive procedures bring this specialty closer to a surgery practice in contrast with other internal medicine subspecialties [10]. Nurses from the gastroenterology medical department assist physicians in all high-risk diagnostic and therapeutic procedures, with a high risk of adverse events, having similar job demands with nurses from the surgical departments. Additionally, they provide care for patients after interventions and for patients in need of extensive care (such as cirrhotic or oncological patients).

Burnout is a process in which the psychological resources of an employee are gradually depleted as a consequence of prolonged stress at work [4]. A mismatch between the resources of the worker and the job demands may lead to burnout if the individual’s coping mechanisms and skills are dysfunctional [14]. Research has found that work-related variables (high workload [15,16], working in shifts [17], as well as sociodemographic factors (age, marital status, and gender [18]) increase the risk of burnout.

Although burnout can be correlated with job dissatisfaction, fatigue, occupational stress, and depression [15,19], it may be present in their absence or absent in their presence [20]. Classified in the ICD-11 as an occupational phenomenon, burnout cannot be considered a medical condition [21] and is a distinct entity from depression. The emotional exhaustion dimension of burnout has been suggested to correlate more closely to depression or other psychological disorders than depersonalization and low personal accomplishment [22,23].

There are conflicting research findings as to whether the appearance of burnout syndrome should be attributed to the type of patient or to the continuous demands that arise from hospitalized patient care [24,25].

For effective prevention of this syndrome, it is most important to identify the occupational, sociodemographic, and psychological variables related to its development [26,27,28]. Identifying the risk factors involved, with solitary or combined action, is an essential element that can contribute to the design and implementation of prevention programs or interventions for nurses at risk, thus avoiding the effects that burnout could have on nurses’ professional and personal lives, patient care, and hospital efficiency.

The objectives of our study are to measure the burnout levels in GE nurses, to assess the possible differences that can occur depending on the department that they work in, to identify and explore occupational or lifestyle-related risk factors associated with burnout and to identify possible protective factors which could be considered for prevention programs.

## 2. Materials and Methods

This study was part of the occupational health periodical medical examination, which is mandatory for healthcare employees according to national legislation and is performed once every 12 months.

From January to March 2019, during the occupational health check-up for the year 2019, the nurses of the surgical and medical department of a gastroenterology unit of a healthcare providing institute were asked to complete two questionnaires in paper and pencil format, before or after the mandatory occupational health assessment. The answers were anonymous and the occupational health physician who was responsible for medical examinations was present and could bring clarifications regarding questions at any time

Participation was voluntary, with each participant completing and signing an informed consent form. The approval from the ethics committee was not mandatory because the assessment of burnout was part of the periodical occupational health examination.

We included all the subjects who agreed to fill in the questionnaires in this study. Altogether, from a total of 111 nurses working in the gastroenterology unit and asked to participate, 76 nurses completed the questionnaires in paper and pencil format, 45 from the medical department, and 31 from the surgical department, with a response rate of 68.5 percent. Subjects who had more than two missing values for the entire questionnaire were subsequently excluded (five subjects in the medical department and one in the surgical department). For those with a maximum of two values missing for the entire questionnaire, we replaced the missing value with the mean of the subject’s existing values for the subscale in question.

We assessed job demands such as shift work, workload, and length of service as psychosocial risks of the work environment. The data was collected together with sociodemographic information (age, gender, marital status), smoking status, physical activity outside work, and medical history of chronic illnesses, through a simple questionnaire that we developed, with open-ended questions. Burnout was measured using an adapted version of the Maslach Burnout Inventory questionnaire with a 5-level frequency scale, developed by Balgiu Beatrice in 2010 [29], suitable for paper and pencil completion, mainly because of the poor computer skills of the subjects. The emotional exhaustion subscale comprised nine items, the depersonalization subscale six items, and the personal accomplishment subscale ten items. An example item for the emotional exhaustion (EE) subscale is “I feel emotionally drained”, for the depersonalization (DP) subscale is “Sometimes I feel indifferent regarding what happens with my colleagues or my subordinates” and for the personal accomplishment (PA) subscale is “I can find the right solution in conflict situations.” The items are scored on a 5-level frequency scale ranging from 1 (very rarely) to 5 (very often). Most of the items for the personal accomplishment subscale (except one) had a reversed quotation.

In order to assess the level of burnout. we calculated a sum score of the dimensional scores. The reference values applied in order to assess the level of burnout on the three dimensions were: for emotional exhaustion (low: 9–18, moderate: 19–27, high: 28–45), for depersonalization (low: 6–12, moderate: 13–18, high: 19–30) and for personal accomplishment (low: 10–20, moderate: 21–30, high: 31–50).

We performed a descriptive analytical cross-sectional study that is useful to establish preliminary evidence for a causal relationship and for examining the associations between exposure and a negative health outcome, where there is a lack of information on the time of onset, as in our population sample.

The results were expressed using descriptive statistics as either: mean (standard deviation) for continuous data or percentages and counts, for the categorical variables. To verify the association between the occurrence of burnout and the categorical variables, we applied Fisher’s exact test. This test was chosen due to the relatively low number of subjects in the studied categories, especially those involving a high risk of burnout. Such a low number would constitute a limit for the Chi-squared test, which is usually performed to compare proportions but does not hinder the results of the Fisher’s exact test. The strength of the association between the variables was evaluated by the Odds Ratio (OR), with the corresponding confidence interval (CI). The margin of error used in the statistical test decision was α < 0.05 and the confidence level was 95%. The statistical calculations were performed using Origin Pro 2019 Software.

## 3. Results

### 3.1. Characteristics of the Study Sample

According to our inclusion criteria, stated above, the study sample consisted of 70 nurses, 30 from the surgical department (SD), and 40 from the medical department (MD) of the gastroenterology unit. The subjects were all females, with a mean (standard deviation) age of 42.24 (8.89) in the SD vs. 40.24 (8.55) in the MD, most of them were married (73%, N = 22 for SD, 78%, N = 31 for MD), they worked in shifts (96% N = 29 for SD; 83%, N = 33 for MD), they had an average workload (patients attended per day) of 11.9 (2.96) in the SD vs. 18.45 (8.18) in the MD, a length of service on average of 12.8 (11.86) years in the SD and of 11.39 (10.64) years in the MD. Most of the nurses were non-smokers (86%, N = 26 for SD; 75%, N = 30 for MD),were physically active outside work (60%, N = 18 for SD; 64%, N = 26 for MD) and had no chronic illnesses (73%, N = 22 for SD; 78%, N = 31 for MD).

### 3.2. The Burnout Assessment

**Figure 1:**
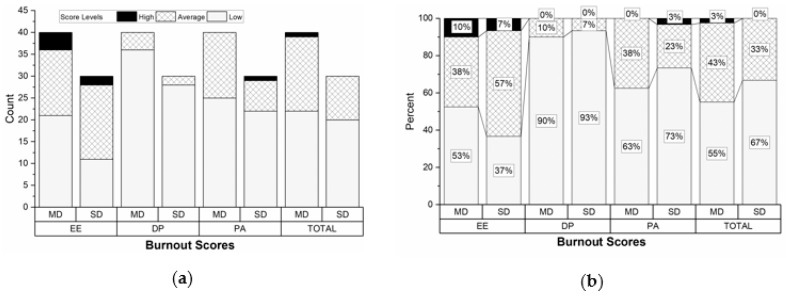
The distribution of the burnout scores according to the three burnout dimensions and department (MD = medical department; SD = surgical department): (**a**) Illustrated as number of cases; (**b**) illustrated as percentages.

Regarding the burnout assessment, we did not have significant differences between the two departments, as shown in Figure 1. In the SD, the total burnout score was low for 67% (N = 20) of the nurses and moderate for 33% (N = 10). The nurses in this department had the lowest burnout scoring, except for the EE dimension, with 64% (N = 19) cases of moderate and high scoring. In the MD, most nurses had low scores for all three burnout dimensions, but in this department, there were a significant number of cases with moderate scoring, a few cases with high scoring for the EE category, and one nurse had a high total score.

**Figure 2:**
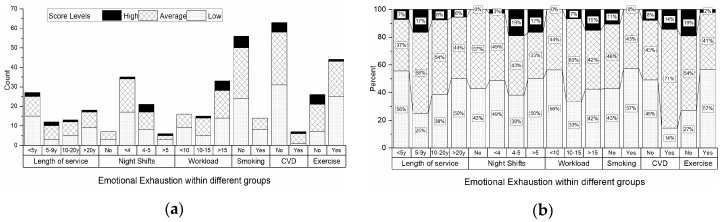
The distribution of the emotional exhaustion burnout scores according to work-related risk factors, lifestyle-related risk factors, and the health status for all the participants: (**a**) Illustrated as number of cases; (**b**) illustrated as percentages.

According to the results illustrated in Figure 2, most nurses (both from the medical and surgical department) with high burnout levels for the EE subscale had a length of service higher than five years, performed over four night shifts per month, had a workload of more than ten patients per day, were non-smokers and did not perform physical activities outside work. Regarding their health status, 14% (N = 1) of the nurses with high EE scores had a known diagnosis of cardiovascular disease (CVD), but there were also nurses with high EE scores and no CVD (8%, N = 5).

### 3.3. Associations between Individual Study Variables and Burnout Levels

In Table 1 we assessed the relation between different risk factors, as individual variables, and burnout levels. We found a significantly high association between high burnout levels for the EE subscale together with performance of more than four night shifts/month and a lack of physical activity outside work. The length of service, smoking habit, and having CVD were not related to high burnout levels.

### 3.4. Associations between Combinations of Different Variables and Burnout Levels

In a more complex analysis of combined risk factors versus burnout scores, strong associations could be found. A variety of combinations between work-related risk factors (such as workload, night shift), lifestyle-related risk factors (exercise), and health status (CVD) were found significantly more often in the high EE risk group, as shown in Table 2. The strongest associations regarded the lack of exercise outside work together with either the performance of more than four night shifts/month or an increased workload (more than 15 patients/day).

## 4. Discussion

The purpose of this study was to identify and explore factors associated with a high risk of burnout among gastroenterology nurses. As they work in two different departments (medical and surgical departments) of a gastroenterology unit, at first we expected to find many differences between the two groups mainly because of the different job demands, but even in the preliminary analysis of our results, we saw many similarities regarding burnout syndrome. This can be explained by the fact that the gastroenterology medical department job requirements are not very different than those of the surgical department, due to the many invasive procedures performed on a daily basis, as highlighted by other authors [10].

Our group of nurses was an all-female group. This is consistent with the preference of women for healthcare and occupations involving care, stated by different studies [30,31]. Although there is a strong predominance of women in this professional category, it is not possible to ignore the influence of gender on burnout [31]. Regarding age, we did not find any significant associations with high levels of burnout, even if our average age, 42.24 in the SD and 40.24 in the MD, is included in an age interval that is considered to be most vulnerable by some authors [32,33,34]. This is consistent with other studies that believe that younger nurses are at greater risk of burnout [35,36,37]. With respect to the influence of marital status, even if most of our subjects in both departments were married, results did not indicate what many studies highlight, which is that social and family support provide protective influence [38,39,40].

The participants from both departments showed high levels of emotional exhaustion, consistent with the results of other studies which state that EE is the most common dimension of burnout [34,35,41,42,43,44,45,46,47,48,49]. Additionally, for this form of burnout, we had the lowest number of low scoring, with important percentages for moderate scoring (57% in the SD and 38% in the MD).

Nurses working in hospital units have an increased workload, and they work in shifts, which can lead to work stress and increased EE [50]. Occupational variables associated with burnout include working night shifts [37,51] and length of service or seniority [6].

At first, trying to explore the known associations between occupational risk factors and burnout, we could see that high EE scores were significantly associated with working more than four night shifts per month. In addition, we found a strong association between burnout and the lack of physical activity outside work, a risk factor that concerns the lifestyle habits of nurses. This last finding is not consistent with other studies [52,53].

Considering our previous indication that work-related risk factors, as well as lifestyle-related risk factors, are involved in the high levels of emotional exhaustion in gastroenterology (GE) nurses, we wanted to assess the outcome of being exposed to a combination of risk factors.

What we found when we studied the combined factors’ influence was that high levels of EE can occur in a wide variety of associations between occupational and non-occupational risk factors. Nurses that did not perform physical activity outside work and had an increased workload and/or carried out more than four night shifts per week had a significantly higher risk of burnout. The occupational risk represented by the increased workload did not significantly influence the nurses’ levels of EE, but it became a significant hazard once associated with working over four night shift per month, with or without exercising, and with or without having a chronic illness, such as cardiovascular diseases. The night shift had a strong, significant influence on the levels of burnout on its own, as well as combined with an increased workload, a lack of exercise, or the presence of cardiovascular diseases. The length of service, on average, of 12.8 years in the SD and of 11.39 years in the MD did not have a significant influence on the levels of burnout. This effect can be attributed to the small number of senior workers in our study, considering the findings by other authors in which seniority represents a significant risk factor for burnout [6].

According to our findings, work-related risk factors have been indicated as the main trigger for burnout in GE nurses. The lack of medical personnel is also affecting healthcare professionals, increasing the workload and the need to perform more night shifts for hospital employees.

Preventive actions are needed in order to decrease the risk of burnout. From our results, we can state that physical activity outside work could be a protective factor since most of the subjects at high risk of burnout were lacking exercise. Interestingly, none of the nurses who smoked were part of the high EE risk group. This could be attributed to the fact that smoking implies more work breaks and may be considered a relaxing activity.

The main contributions of our research consist of highlighting a research gap regarding the importance of burnout assessment in gastroenterology nurses, together with identifying protective factors and associations of occupational and non-occupational risk factors involved.

This study has limitations. (1) The main limitation consists of the small sample of nurses involved, which may have influenced the results. Nevertheless, it should be kept in mind that this was a representative sample of the population of nurses working in gastroenterology units of hospitals. In addition, it was a varied sample of professionals, working in two important and different hospital departments. (2) Due to the descriptive, cross-sectional study design, the results cannot be considered very strong, but they can represent a good starting point for further investigations. (3) We did not have enough control of confounding variables, such as predominance of women, affecting other additional variables.

Our research could have relevant implications for society and policymakers and be a starting point for future research. For example, studies that further explore the prevalence of burnout in gastroenterology units, with a larger nurses’ study population; comparative studies analyzing the relation between GE physicians and nurses’ burnout levels; or studies that assess the effectiveness of interventions for burnout prevention, adding exercise to other well-established means, such as mindfulness, meditation, or coping programs [54].

## 5. Conclusions

Gastroenterology nurses, from both surgical and medical practice, are similarly affected mostly by high levels of EE, which could be attributed to a large number of invasive procedures specific to the medical gastroenterology department.

Work-related risk factors, such as an increased workload, alternate working schedule with a large number of night shifts, together with a sedentary lifestyle expressed as a lack of physical activity outside work, and the presence of cardiovascular diseases, result in a greater prevalence of burnout.

In our study, smoking appeared as a “protective factor” for burnout, maybe because this habit is perceived as a relaxing activity and it implies taking more frequent breaks from occupational demands. In this category of healthcare workers, preventive actions are needed. As it appears from our results, physical activity outside work could be a protective factor for burnout, and thus an exercise program could boost the effectiveness of well-established means of burnout intervention programs.

Future research should assess the effectiveness of exercise in addition to other well-established means of burnout interventions and compare the burnout levels of GE nurses with those already studied among GE physicians.

## Figures and Tables

**Table 1:** Associations between individual study variables and burnout levels.

Variables	Low and Moderate Burnout Levels *n* (%)		High Burnout Levels *n (%)*		OR	95% CI	*p* *
	No	Yes	No	Yes			
**Work-related risk factors**							
Length of service 5–9 years	54 (77.14)	10 (14.28)	4 (5.71)	2 (2.85)	2.7	0.43–16.77	0.271
Night shifts > 4/month	42 (60)	22 (31.43)	1 (1.43)	5 (7.14)	9.55	1.05–86.85	0.028
Workload > 15 patients/day	36 (51.42)	28 (40)	1 (1.43)	5 (7.14)	6.43	0.71–58.19	0.075
**Lifestyle-related risk factors**							
Smoking habit	50 (71.43)	14 (20)	6 (8.57)	0 (0)	0	0	0.247
No Exercise	43 (61.43)	21 (30)	1 (1.43)	5 (7.14)	10.24	1.12–93.29	0.023
**Risk factors associated with the health status**							
Cardiovascular diseases	58 (82.86)	6 (8.57)	5 (7.14)	1 (1.43)	1.93	0.19–19.39	0.481

* According to Fisher’s Exact Test.

**Table 2:** Associations between combinations of different variables and the burnout levels.

Variables	Low and Moderate Burnout Levels *n* (%)		High Burnout Levels *n* (%)		OR	95% CI	*p* *
	No	Yes	No	Yes			
**Combined risk factors**							
Workload >15 patients/day and No exercise	54 (77.14)	10 (14.29)	2 (2.86)	4 (5.71)	10.8	1.74–67.1	0.012
Workload >15 patients/day and Night shifts > 4 /month	49 (70)	15 (21.43)	2 (2.86)	4 (5.71)	6.53	1.08–36.25	0.042
Workload >15 patients/day or Night shifts > 4 /month	29 (41.43)	35 (50)	0 (0)	6 (8.57)	-	-	0.034
Night shifts > 4 /month and No exercise	59 (84.29)	5 (7.14)	1 (1.43)	5 (7.14)	59	5.72–608.25	<0.001
(Workload >15 patients/day or Night shifts > 4/month) and No exercise	53 (75.71)	11 (15.71)	1 (1.43)	5 (7.14)	24.09	2.55–226.99	0.001
No exercise or Cardiovascular diseases	40 (57.14)	24 (34.29)	1 (1.43)	5 (7.14)	8.33	0.91–75.65	0.040
(Workload >15 patients/day or Night shifts > 4/month) and (No exercise or Cardiovascular diseases)	52 (74.29)	12 (17.14)	1 (1.43)	5 (7.14)	21.66	2.31–202.89	0.002
Night shifts > 4/month and (No exercise or Cardiovascular diseases)	59 (84.29)	5 (7.14)	1 (1.43)	5 (7.14)	59	5.72–608.25	<0.001

* According to Fisher’s Exact Test.
